# Improvement in nutritional quality of traditional unleavened flat bread using Quality Protein Maize

**DOI:** 10.3389/fnut.2022.963368

**Published:** 2022-11-23

**Authors:** Navjot Kaur, Ramesh Kumar, Alla Singh, D. Shobha, Abhijit Kumar Das, Dharampaul Chaudhary, Yashmeet Kaur, Pardeep Kumar, Priti Sharma, Baljit Singh

**Affiliations:** ^1^ICAR-Indian Institute of Maize Research, Ludhiana, India; ^2^AICRP on Post Harvest Engineering and Technology, University of Agricultural Sciences, Bengaluru, India; ^3^School of Agricultural Biotechnology, Punjab Agricultural University, Ludhiana, India; ^4^Department of Food Science and Technology, Punjab Agricultural University, Ludhiana, India

**Keywords:** Quality Protein Maize, landrace, mineral content, amino acid, unleavened flat bread, Indian traditional flat bread, *chapatti*

## Abstract

Maize grains are consumed majorly in the form of unleavened flat bread (*chapatti*) in the South East Asian region. The landraces are better accepted for their *chapatti*-making attributes such as grain color and good organoleptic properties. However, these cultivars are low in essential amino acids, particularly lysine and tryptophan content. Hence, an investigation was performed to identify maize genotypes with high nutritional value coupled with good *chapatti*-making qualities. Seven genotypes, comprising two Quality Protein Maize (QPM) hybrids, two normal maize hybrids, and three normal white maize landraces were assessed for their physical characteristics, proximate composition, and *chapatti*-making quality. Landrace 593 showed the highest protein and ash content. Flours obtained from different genotypes were significantly different (*p* ≤ 0.001) in terms of protein content, color value, textural, as well as mineral content. PMH 10 and IQMH 203 exhibited the highest and lowest hydration index, respectively. Two QPM hybrids showed significantly higher lysine and tryptophan content as compared to other genotypes. QPM hybrids were identified as the promising material with improved nutritional quality with respect to *chapatti* making. In combination with mustard greens, maize chapatti constitutes an important traditional delicacy in north India. The enhanced nutritional quality of QPM *chapattis* is an added advantage. We show the differentiation of *chapattis* made from QPM and normal maize using a rapid protocol developed previously. This is expected to enable the development and quality control of commercial enterprises based on high protein quality QPM.

## Introduction

In terms of production, maize is the most important globally and the third most important cereal in India.^1^ It is regarded as good for health due to its nutraceutical properties. Celiac disease is an autoimmune chronic illness characterized by small intestine inflammation and villous atrophy (1). Patients with Celiac disease are advised to take gluten-free diets. Therefore, cereal grains such as wheat and barley were excluded from the diet of patients with celiac disease (2, 3). However, it is challenging to adhere to a restrictive gluten-free diet due to various reasons. First, the choice of food becomes limited because cereal products play predominant roles in a daily diet. Second, most processed foods contain gluten-based products as a major or an additional component (4). Moreover, the replacement of gluten is also a technological challenge, as the absence of gluten exhibits quality deficiencies such as poor expansion, color, and texture in final products (5, 6). Hence, the production of gluten-free foods possessing high nutritional value and consumer acceptance can be of immense health benefit to patients with celiac disease.

Maize is one of the preferred gluten-free cereal grains, with suitability to prepare food products mainly addressed to patients with celiac disease (7). Maize flour is consumed as food (35%), mainly in the form of unleavened flat bread (‘‘*chapatti*,’’ also known by the name ‘‘*roti’’*), in South East Asia. In northern parts of India, especially in the state of Punjab, the combination of maize *chapatti* with mustard green is a very popular traditional dish. Government of Punjab, India, has listed this traditional delicacy in its culture section and has mentioned the availability of entrepreneurial opportunities in cuisine.^2^ However, maize is limited in terms of its nutritional properties as being low in essential amino acids such as lysine and tryptophan, which leads to protein-energy malnutrition (5). If biofortified maize is utilized for making traditional delicacies, it would provide the benefits of improved gluten-free, amino acid nutrition. However, the sensory quality and nutritional attributes subsequent to product development need to be ascertained to evaluate its potential deployment.

To overcome malnutrition, the fortification of staple foods such as flatbread was considered (8). To minimize the requirement and to cut the cost of fortification, quality protein maize (QPM) has received much attention owing to its well-balanced protein and also being gluten-free grain, which reduces the risk of various diseases. QPM flour can be used to prepare nutrition-enriched *chapatti* with improved amino acid balances, which can help to overcome the national protein-calorie malnutrition problem (9). Incorporation of QPM flour in common food systems is expected to add value to it, and also provide convenient substitutes to expensive nutritious foods, with the changing lifestyles and trends around the world.

*Chapatti* has served as a staple diet to a majority of households in India, Pakistan, and some parts of the Middle East (10). Traditionally, it is prepared from wheat flour dough after rolling into a circular sheet followed by baking of both sides at high temperatures for a short time duration, which results in the puffing of *chapatti* by rapid steam formation. The major protein (gluten) present in wheat possesses unique properties to form a cohesive dough, which can trap gases and also enable mechanical sheeting but is not tolerated by patients with celiac disease. Although maize flour is healthy and gluten-free, the absence of gluten results in weak dough-binding properties and affects the *chapatti*-making quality (6). The dough behavior, rheological properties, and sensory qualities such as color, flavor, texture, and aroma of *chapatti* directly affect the acceptability of *chapatti* (11). In India, most of the population consuming maize as food prefers locally available maize landrace for *chapatti* due to its fine texture and unique taste. Keeping in view the preference of people and nutritional aspects, the present study was conducted to evaluate *chapattis* made from different types of maize genotypes, *viz*., landraces, normal, and QPM hybrids. Overall, the study aimed to compare the *chapatti*-making ability and nutritional quality of seven maize genotypes.

## Materials and methods

### Materials

The materials consist of grains of seven maize genotypes. These genotypes represented both white and yellow maize including landraces, normal hybrids, as well as QPM hybrids. Various genotypes were grown at ICAR-IIMR Ladhowal farm, Ludhiana, Punjab. The genotypes were harvested in October 2020 and dried properly followed by storage in airtight containers at ambient temperature. A sample from each genotype was selected randomly. The details of the genotypes selected for the study are as follows:

**Table:** 

Sr. No.	Variety	Hybrid	Developing Organization
1	PMH 10	Normal Orange Maize Hybrid	PAU, Ludhiana
2	IQPMH 1708	QPM Experimental Hybrid	ICAR-IIMR, Ludhiana
3	IQMH 203	QPM Hybrid	ICAR-IIMR, Ludhiana
4	MCFL 15	Normal White Maize Landrace	ICAR-IIMR, Ludhiana
5	MCFL 346	Normal White Maize Landrace	ICAR-IIMR, Ludhiana
6	White Hybrid 574	Normal White Maize Experimental Hybrid	ICAR-IIMR, Ludhiana
7	Landrace 593	Normal White Maize Landrace	ICAR-IIMR, Ludhiana

All the grains were screened to remove extraneous matter. The cleaned grains were stored in sealed packages at room temperature. Each genotype was assessed for its physical characteristics and was ground to make maize flour (<200 μ) using a laboratory mill (Perten Instruments, Hagersten, Sweden), sieved, and packed for further analysis and processing.

### Analysis of maize kernel, maize flour, and *chapatti* was performed by following methods

#### Assessment of physical properties of maize kernels

Maize genotypes were assessed for their physical characteristics such as kernel type (flint, dent) and kernel color (white, orange, and yellow), as well as other physical and quality parameters described below.

##### Thousand kernel weight

Thousand kernel weight was noted by weighing a hundred grains on an electronic weighing balance and multiplied by 10 and results were expressed in grams (g).

##### Specific gravity

A measuring cylinder (100 ml) was filled with water up to a mark. Pre-weighed corn grains were poured into the cylinder and a rise in the volume of water was noted.

##### Linear dimensions

The linear dimensions (in triplicates) such as length (L), breadth (b), and thickness (t) of the corn kernel were measured by a vernier caliper (12).

##### Shape index

The shape index is a measure of the kernel shape that is oval or spherical. The data are computed according to the following equation:


(1) $$S\,h\,a\,p\,e\,I\,n\,d\,e\,x=\frac{l}{\sqrt{b}\,X\,t}$$

where, b = breadth and t = thickness.

If the shape index is greater than 1.5, the kernel is considered oval and if it is less than 1.5, the kernel will be of spherical shape (13).

##### Hydration capacity (%) and hydration index

Hydration capacity and hydration index were determined according to the method described by Williams et al. (14). To measure hydration capacity, a known weight of grains is transferred into a beaker containing water. Beaker was covered with aluminum foil and left overnight at room temperature. On the next day, the water was drained and the weight of wet grains was noted and calculated as follows:


(2) $$\begin{array}{c} HydrationCapacity\left(\%\right)\\ =\frac{W\,e\,i\,g\,h\,t\,a\,f\,t\,e\,r\,S\,o\,a\,k\,i\,n\,g-W\,e\,i\,g\,h\,t\,b\,e\,f\,o\,r\,e\,S\,o\,a\,k\,i\,n\,g}{W\,e\,i\,g\,h\,t\,o\,f\,S\,e\,e\,d\,s} \end{array}$$

(3) $$H\,y\,d\,r\,a\,t\,i\,o\,n\,I\,n\,d\,e\,x=\frac{H\,y\,d\,r\,a\,t\,i\,o\,n\,C\,a\,p\,a\,c\,i\,t\,y\,p\,e\,r\,S\,e\,e\,d}{W\,e\,i\,g\,h\,t\,o\,f\,o\,n\,e\,s\,e\,e\,d}$$

### Analysis of maize flour

#### Proximate analysis

Proximate composition of maize flour was determined using the standard method (15).

##### Moisture content

The moisture content of the flour was analyzed by the hot air oven method after drying at 100°C for 2 h and the percent moisture content is calculated from loss in moisture from the sample (15).

##### Fat content

Fat content of the flour samples was analyzed by FOSS instrument-Soxtec 2045 (Sweden). Approximately 2 g of flour sample was added in a thimble followed by the addition of petroleum ether (70 ml) in pre-weighed extraction beakers. The instrument was pre-heated prior to analysis at a temperature of 130–135°C. After a pre-determined temperature, extraction beakers were attached and allowed to boil for 20 min followed by rinsing for 20 min. After the solvent was recovered for 10 min, the extraction beakers were removed and weighed after cooling at room temperature. Crude fat (%) was calculated from the increase in the weight of the extraction beaker (15).

##### Protein content

The protein content of flour samples was determined by the micro-Kjeldahl method. The macro-Kjeldhal method was used to determine the nitrogen content for all raw materials (15). A general composite conversion factor of 6.25 was used to calculate the percent crude protein content.

##### Ash content

The sample was taken in pre-weighed crucibles followed by charring at a hot plate until no fumes come out. Charred samples were placed in a muffle furnace at 550°C for 5 h and were then placed in the desiccator. The weight of the final crucible is noted as ash content (15).

##### Carbohydrate content

Carbohydrate content was calculated using a subtraction method, that is, 100 – moisture, ash, fat, and protein contents.

#### Pasting properties of maize flour

The pasting properties of the maize flour samples were determined by using the Rapid Visco Analyzer (RVA) model starch Master (Newport Scientific, Warrie Wood, Australia). The operation procedure is followed as given below: The RVA was allowed to warm up for 30 min prior to the experiment. The pre-weighed sample was poured into a canister followed by the addition of water (25 ml). The paddle was inserted into the canister and vigorously shaken up and down 10 times through the sample until it mixes properly. Insert the canister into the pre-adjusted instrument. The programmed heating and cooling cycle were given. After the completion of the test, the pasting properties such as peak viscosity, final viscosity, breakdown, and setback were noted. The canister was removed from the instrument and the sample was discarded.

#### Mineral estimation

The mineral content of maize flour was determined for five different minerals viz. Fe, Zn, Ca, Mg, and K using the OptimaTM2100DV Spectrometer (Perkin Elmer). The mineral concentrations were recorded as ppm, which can be represented as mg of mineral per 100 g of sample.

#### Amino acid analysis

Amino acid analysis (tryptophan and lysine) of the maize flour samples was carried out by following a previously described method (16).

#### Color analysis

Color analysis of flour samples was carried out using a Hunter lab colorimeter on the basis of L*, a*, and b* values. The colorimeter was calibrated with the standard black and white plate to set zero. The samples were uniformly packed in clean petri plates. The different places on the surface were given three exposures by the colorimeter. Readings were displayed as a*, b*, and L* where the ‘a’ value indicates the redness to greenness, the ‘b’ value measures the blueness to yellowness, while the ‘L’ value ranges from 0 (black) to 100 (white) which indicates the measure of lightness (17).

### *Chapatti*-making

#### Preparation of *chapatti*

**FIGURE 1:**
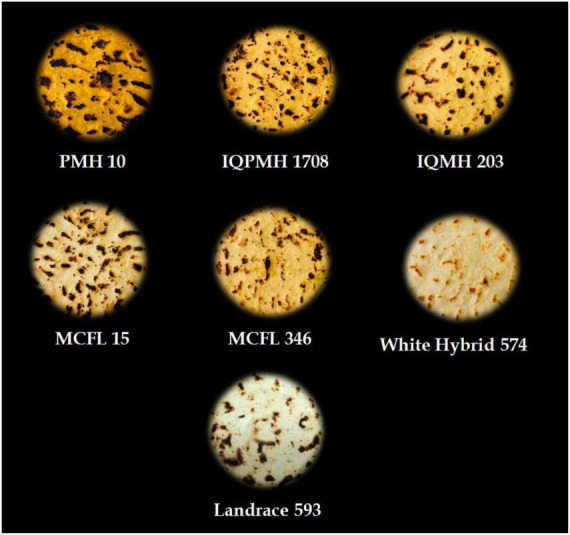
*Chapattis* prepared from different genotypes.

*Chapatti* was prepared by adopting the method as described by previous researchers with slight modifications (18). Corn flour was mixed with an optimum amount of lukewarm water to form a smooth dough. Dough balls of similar weight were prepared, placed on a rolling board, and round sheeted using a rolling pin to make *chapatti*. The raw *chapatti* was immediately placed on a hot plate (tawa) and baked at 220°C on one side and then on the other side. It was again turned until fully baked. The *chapattis* prepared from different genotypes (Figure 1) were allowed to cool for 10 min at 25°C and then packed in polythene pouches and placed in an airtight container for further analysis.

#### Physico-chemical properties of *chapatti*

##### Water absorption capacity

The water absorption capacity of maize flour to form dough was measured by employing the method outlined by Gujral and Gaur (19). The calculated amount of water was added to the flour (200 g) to form the smooth and non-stick dough, appropriate for sheeting without exhibiting any cracks. Then, the optimum amount of water added was noted.

##### Texture analysis of *chapatti*

A strip of each *chapatti* was tested (in triplicates) for Shear value and Texture Profile Analysis (TPA) on the TA/XT2 Texture analyzer (Stable Micro Systems, Surrey, England) by following the method described below.

**Texture Profile Analysis (TPA):** Texture Profile Analysis parameters including adhesiveness, cohesiveness, springiness, hardness, chewiness, and gumminess were measured. Samples were cut into uniform sizes and a cylindrical aluminum (P25) probe was used to exert pressure. The instrumental condition used is as follows: Pre-test speed: 10.0 mm/min, Post-test speed: 10.0 mm/min Trigger: 15.0 g, Load cell: 20.0 kg (20).

**Shear Value:** Shear value was measured by cutting the strip (4 cm × 2 cm) of *chapatti* (taken from the center of the *chapatti*) using Warner Bratzler Blade (HDP/BSK). The following conditions were employed: load cell—50 kg, target mode distance—4.5 mm, pre-test speed—1 mm/s, test speed—2 mm/s, post-test speed—10 mm/s, and trigger force—10 g. The force required to shear the strip of *chapatti* into two pieces was noted. Three measurements were taken for each sample in triplicates and average values are reported (21).

##### Proximate, amino acid, and mineral content of *chapatti*

*Chapatti* was analyzed for proximate composition, amino acid content, mineral content, and color as per the previously described methods for flour.

##### Sensory evaluation

*Chapatti* prepared from each genotype were analyzed for sensory scores in terms of color, appearance, taste, mouth feel, and overall acceptability in order to find the best genotype for the development of chapatti. Semi-trained and untrained panelists were selected to evaluate the *Chapatti*. *Chapatti* was placed on white paper and labeled with numbers to avoid any bias. A total of 10 semi-trained panelists (five men and five women, between the age group of 25 and 55 years) were selected for sensory evaluation. All the panelists were instructed to rinse their mouths properly with water after tasting every sample and to score the chapatti samples based on the acceptance. A 9-point hedonic scale presenting a score of 1 for extremely disliking and 9 for extremely liking was used. The final score was calculated by averaging the scores provided by all the panelists (22).

##### Rapid differentiation

**FIGURE 2:**
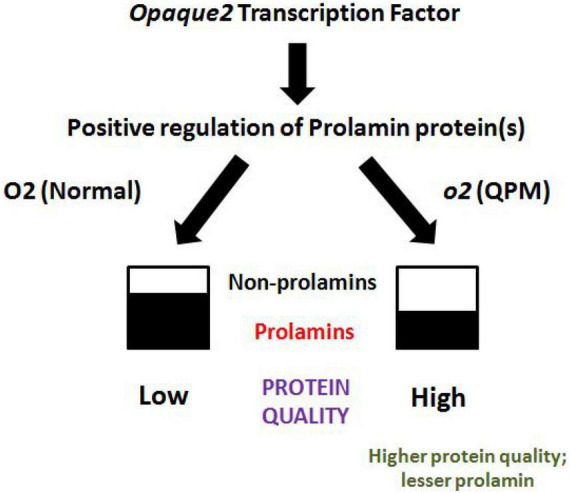
Protein quality in normal maize and QPM. By virtue of its replacement by higher quality non-prolamins, the lower expression of prolamins increases protein quality on one hand and decreases the chances of any adverse reactions in some patients with celiac disease as observed in normal maize.

A process was designed to rapidly differentiate normal maize grains from QPM grains utilizing molecular differences in the two groups (Figure 2). An Indian Patent application (No. 202211015547) has been filed for this process. The same process was used to differentiate between the normal maize *chapattis* from QPM *chapattis*. The method records OD at 595 nm for nutritionally poor protein to act as a proxy for maize protein quality.

### Statistical analysis

Data were recorded in triplicates and presented as mean ± standard deviation. The data were analyzed using SAS version 9.4 software. The least significant difference (LSD) was used as the test for significance for different measured traits among the treatments/genotypes. Paired ‘t’ test was used to test the significant changes in different attributes between maize raw flour and *chapatti* made out of it.

## Results and discussion

The physical properties of maize are important for milling and processing industries which usually prefer large grains. The greater the size of grains the more would be the extraction of starch and oil (23).

### Physical and dimensional properties of maize kernels

The physical characteristics such as color, grain type, dimensions, thousand kernel weight (TKW), specific gravity, hydration capacity, hydration index, and shape index of different genotypes have been mentioned in Table 1. Each of the maize genotypes recorded significantly different thousand kernel weights (TKWs). It was observed that the kernel weight of White Hybrid 574 was highest (368.47 g) followed by Landrace 593 (356.43 g), MCFL 346 (322.33 g), MCFL 15 (298.83 g), and PMH 10 (291.66 g). Karthik et al. (13) reported that the TKW of different maize genotypes ranged from 80.50 to 321.85 g, which is in agreement with the present study. Maize genotypes having TKW greater than 290 gm are appropriate for industrial applications because they provide high yields in different products (23). The dimensions such as length, breadth, and thickness of various corn genotypes varied significantly (*p* ≤ 0.001) between 9.45 and 11.28 mm, 7.20 and 9.53 mm, and 3.46 and 5.56 mm, respectively. QPM (IQPMH 1708) genotype had the smallest grain size out of the seven genotypes under study. The Thousand Kernel Weight (TKW) of QPM hybrids (HQPM 1 and HQPM 7) was observed to be in the range of 275.5 and 288.3 g by Sangeeta and Grewal (24). The shape index is important in determining the productivity of various genotypes as flat grains are considered desirable grain quality to meet the requirement of high productivity (25). The data showed that two genotypes namely MCFL 15 and MCFL 346 were of spherical shape whereas White Hybrid 574 and IQPMH 1708 showed no significant difference in shape index and were oval-shaped with a higher yield. The study of Srinivas et al. (25) stated that the factors contributing to shaping variation could be the position of grain on the cob, varietal or environmental difference, and distorted or twisted pattern of rows within the cobs. The results of Bolade (26) with respect to TKW, length, and width of the maize ranged from 223.7 to 284.2 g, 9.1 to 11.9 mm, and 8.1 to 9.5 mm, respectively.

**TABLE 1:** Physical and dimensional properties of maize kernels from different genotypes.

	Genotypes	Type	Color	Normal/Opaque	Length (mm)	Breadth (mm)	Thickness (mm)	TKW (gm)	Specific gravity	Hydration index	Shape index
1	PMH 10	Flint	Orange	Normal	11.06 ± 0.48^A^	8.51 ± 0.35^B^	4.41 ± 0.33^B^	291.66 ± 0.50^E^	0.353 ± 0.004^C^	0.432 ± 0.002^A^	1.81 ± 0.07^AB^
2	IQPMH 1708	Flint	Orange	Opaque	9.45 ± 0.07^C^	7.2 ± 0.28^D^	3.90 ± 0.29^BC^	196.87 ± 0.45^G^	0.259 ± 0.006^D^	0.401 ± 0.001^B^	1.79 ± 0.08^B^
3	IQMH 203	Flint	Orange	Opaque	9.71 ± 0.10^C^	7.46 ± 0.56^CD^	3.46 ± 0.08^C^	214.20 ± 0.36^F^	0.273 ± 0.002^D^	0.194 ± 0.001^G^	1.91 ± 0.07^A^
4	MCFL 15	Flint	White	Normal	9.77 ± 0.22^C^	9.44 ± 0.31^A^	5.46 ± 0.51^A^	298.83 ± 0.42^D^	0.353 ± 0.004^C^	0.351 ± 0.001^D^	1.36 ± 0.06^D^
5	MCFL 346	Dent	White	Normal	10.50 ± 0.21^B^	9.53 ± 0.25^A^	5.56 ± 0.23^A^	322.33 ± 0.89^C^	0.376 ± 0.001^B^	0.286 ± 0.031^F^	1.44 ± 0.07^CD^
6	White Hybrid 574	Semi-dent	White	Normal	11.28 ± 0.04^A^	7.88 ± 0.22^C^	4.40 ± 0.09^B^	368.47 ± 0.41^A^	0.380 ± 0.001^B^	0.331 ± 0.002^E^	1.92 ± 0.03^A^
7	Landrace 593	Dent	White	Normal	10.39 ± 0.06^B^	9.05 ± 0.14^AB^	5.19 ± 0.32^A^	356.43 ± 0.85^B^	0.404 ± 0.002^A^	0.368 ± 0.001^C^	1.52 ± 0.05^C^
	Means Square				1.46***	2.69***	1.93***	130.32***	0.009***	0.019***	0.160***

Values presented as mean ± standard deviation.

Means in the same column with different superscripts ^abcdefg^ are significantly different (*p* ≤ 0.001).

The means shown in the same column with common superscripts are not significantly different (*p* > 0.05).

TKW, thousand kernel weight.

***Highly significant at 0.001.

The hydration index is a process of water absorption by grains that increases their moisture content and could affect their physicochemical, nutritional, as well as textural properties (27). The hydration index of corn genotypes significantly varied from 0.194 to 0.432 (*p* ≤ 0.001). The hydration index was higher in PMH 10 and a lower value was found in IQMH 203. The lower hydration index might help to extend the shelf life of maize grain during storage (27).

### Pasting properties of maize flour

The pasting properties of flours obtained from seven genotypes are presented in Table 2. A significant difference (*p* ≤ 0.001) was observed for pasting properties, *viz*., peak viscosity (cP), hold viscosity (cP), final viscosity (cP), breakdown (cP), set back (cP), and water absorption capacity (ml) among flours from different maize genotypes depending on the rigidity of starch granules which in turn affect the granule swelling potential (28). Peak viscosity ranged from 207 (IQPMH 1708) to 1,097 cP (PMH 10), indicating the water binding capacity of starch or mixture, which often correlates to the quality of the final product, respectively. The higher peak viscosity may be associated with a high proportion of ungelatinized starch, whereas the lower values might be due to greater degradation through depolymerization and molecular entanglement during processing conditions (29). Breakdown value varied significantly and was higher in MCFL 346 (202 cP) followed by MCFL 15 (117 cP). It is related to the starch response to shear with continuous heating, causing a rupture and resulting in a decrease in viscosity (30). The setback viscosity is related to starch retrogradation and reordering (31) and varied from 908 to 1,696.33 cP. PMH 10 was reported to exhibit a low rate of syneresis and retrogradation of starch molecules (32). The low setback viscosity value of IQPMH 1708 and Landrace 593 flour indicates the lower value of retrogradation. Hence, chapattis prepared from IQPMH 1708 and Landrace 593 genotypes would remain fresh for a longer time (8). Sagbo et al. (33) found the range of peak viscosity and setback viscosity of different maize genotypes varied from 438-1,271.5 cP and 362-2,534 cP, respectively. IQMH-based flour can be used to replace wheat flour for chapatti preparation, which can complement as a source of essential amino acids as well as a gluten-free diet.

**TABLE 2:** Pasting properties and water absorption capacity of maize flours from different genotypes.

	Genotypes	Peak viscosity (cP)	Hold viscosity (cP)	Final viscosity (cP)	Breakdown (cP)	Set back (cP)	Water absorption capacity (ml)
1	PMH 10	1097.00^G^	684.93 ± 1.00^G^	2410.67 ± 2.52^G^	92.00 ± 0.00^G^	1405 ± 2.65^F^	135 ± 2.16^D^
2	IQPMH 1708	207.00 ± 2.65^F^	150.67 ± 2.08^F^	1058.67 ± 4.04^F^	48.67 ± 2.08^E^	908 ± 2.00^E^	146 ± 0.82^C^
3	IQMH 203	403.67 ± 5.03^C^	347.00 ± 4.58^C^	1808.00 ± 5.57^C^	57.00 ± 4.36^D^	1460.67 ± 3.79^C^	135 ± 0.82^D^
4	MCFL 15	603.00 ± 2.65^B^	486.33 ± 0.58^B^	2088.67 ± 4.04^B^	117.00 ± 2.65^B^	1603.33 ± 5.03^B^	153.6 ± 1.25^B^
5	MCFL 346	708.00 ± 5.57^A^	506.33 ± 3.79^A^	2201.67 ± 4.04^A^	202.00 ± 4.58^A^	1696.33 ± 5.13^A^	155 ± 2.94^B^
6	White Hybrid 574	298.00 ± 3.61^D^	283.67 ± 2.52^D^	1336.33 ± 5.13^D^	14.00 ± 2.65^F^	1079.33 ± 4.73^D^	159.3 ± 1.70^A^
7	Landrace 593	251.00 ± 2.65^E^	163.00 ± 3.61^E^	1071.33 ± 2.52^E^	88.00 ± 2.65^C^	908 ± 4.58^E^	126.3 ± 2.05^E^
	Mean Square	175223.09***	103245.32***	1451387.64***	14288.19***	791592.52***	463.44***

Values presented as mean ± standard deviation.

Means in the same column with different alphabets in superscript are significantly different (*p* ≤ 0.001).

The means shown in the same column with common superscripts are not significantly different (*p* > 0.05).

***Highly significant at 0.001.

Water absorption is the addition of lukewarm water to flour to obtain desired consistency of the dough and indicate the baking quality of the flour. A significant difference was observed for the water absorption capacity of different maize genotypes, however, similar water absorption was observed for MCFL 15 and MCFL 346, and PMH 10 and IQMH 203 (Table 2). White hybrid 574 required a higher amount of water (159.3 ml) followed by MCFL 346 (155 ml) for the preparation of dough to make *chapatti*, which could be attributed to the molecular structure of starch, variation in protein content, and presence of high hydrophilic constituents (34). The lowest absorption was found in Landrace 593 (*p* ≤ 0.001). It shows that the genotype White hybrid 574 has a higher ability to retain water during the baking process which provides a desirable soft texture in final products (35). However, the QPM genotypes IQPMH 1708 and IQMH 203 had recorded medium water absorption, i.e., 146 and 135 ml. This indicates that this genotype had soaked a good amount of water which is desirable for the baking of *chapattis*.

### Nutritional composition of maize flour and *chapattis*

Maize genotypes varied significantly with respect to their proximate composition such as moisture, fat, and protein contents (Table 3). The protein content in chapattis was observed to be higher in Landrace 593 followed by MCFL 15. The concentration of protein varied from 6.19 to 8.39% as stated in the previous study conducted by Vaswani et al. (36). MCFL 15 flour had lower moisture (3.02%), and higher ash (1.99%) and crude fiber (1.36%) contents. Sandhu et al. (37) also reported ash, protein, fiber, and carbohydrate contents of 1.66%, 5.18–7.82%, 1.56–2.42%, and 87.6–92.5% for corn flour. The composition of *chapatti* also differed significantly among different genotypes (Table 3). The *chapatti* prepared from genotype IQPMH 1708 showed higher moisture content (31.15%), which is a desirable property to impart softness in *chapattis*, whereas MCFL 15-based *chapatti* had lower moisture content (24.67%). IQMH 203-based *chapattis* were recorded for the highest ash (1.71%) and lowest crude fiber (0.18%) contents. The t-value indicates that there was a highly significant difference between maize flours and *chapatti* for the parameters such as moisture (−21.93), fiber (2.79), and carbohydrate (14.09), whereas fat (−0.79), ash (−2.08), and protein (−0.50) showed no significant difference between flour and *chapatti*.

**TABLE 3:** Proximate composition of maize flour and *Chapatti* from different genotypes.

	Flour	Moisture (%)	Fat (%)	Protein (%)	Ash (%)	Fiber (%)	Carbohydrates (%)
1	PMH 10	5.13 ± 0.01^A^	4.07 ± 0.21^D^	8.08 ± 0.03^E^	1.90 ± 0.16^A^	1.34 ± 0.10^A^	79.48 ± 0.20^C^
2	IQPMH 1708	3.43 ± 0.19^E^	4.57 ± 0.08^B^	8.47 ± 0.05^F^	1.28 ± 0.07^B^	1.10 ± 0.03^B^	81.15 ± 0.10^B^
3	IQMH 203	4.21 ± 0.08^C^	5.13 ± 0.10^A^	8.38 ± 0.54^EF^	1.27 ± 0.10^B^	1.10 ± 0.00^B^	78.62 ± 0.23^C^
4	MCFL 15	3.02 ± 0.08^F^	4.47 ± 0.20^B^	9.58 ± 0.19^C^	1.99 ± 0.14^B^	1.36 ± 0.00^A^	79.58 ± 0.14^C^
5	MCFL 346	3.80 ± 0.06^D^	4.20 ± 0.18^CD^	8.88 ± 0.03^D^	1.24 ± 0.04^B^	1.13 ± 0.01^B^	80.76 ± 0.25^B^
6	White Hybrid 574	4.56 ± 0.09^B^	4.45 ± 0.13^BC^	10.18 ± 0.04^B^	1.08 ± 0.05^B^	1.17 ± 0.00^B^	78.56 ± 0.19^C^
7	Landrace 593	3.44 ± 0.04^E^	4.90 ± 0.10^E^	10.88 ± 0.03^A^	0.45 ± 0.58^C^	1.13 ± 0.01^B^	79.20 ± 0.70^A^
	Mean Square	1.62***	5.83***	0.53***	3.25***	0.03***	8.83***
	***Chapatti***						
1	PMH 10	24.82 ± 0.76^C^	3.93 ± 0.35^B^	8.18 ± 0.06^E^	1.48 ± 0.04^C^	1.06 ± 0.01^B^	60.53 ± 0.51^A^
2	IQPMH 1708	31.15 ± 1.68^A^	4.57 ± 0.08^AB^	8.33 ± 0.32^E^	1.53 ± 0.01^B^	0.98 ± 0.13^B^	53.70 ± 1.64^B^
3	IQMH 203	26.52 ± 3.31^BC^	4.83 ± 0.56^A^	9.63 ± 0.26^C^	1.71 ± 0.01^A^	0.18 ± 0.00^D^	58.93 ± 3.32^A^
4	MCFL 15	24.67 ± 0.52^C^	4.37 ± 0.37^AB^	10.05 ± 0.31^AB^	1.67 ± 0.00^A^	1.26 ± 0.06^A^	59.48 ± 0.93^A^
5	MCFL 346	25.95 ± 2.96^C^	4.00 ± 0.49^B^	8.73 ± 0.02^D^	1.41 ± 0.03^D^	0.99 ± 0.04^B^	58.92 ± 2.49^A^
6	White Hybrid 574	28.27 ± 2.90^ABC^	4.53 ± 0.12^AB^	9.95 ± 0.02^BC^	1.46 ± 0.02^C^	1.02 ± 0.00^B^	54.78 ± 2.93^B^
7	Landrace 593	29.91 ± 0.09^AB^	4.53 ± 0.47^AB^	10.38 ± 0.03^A^	1.36 ± 0.02^E^	0.55 ± 0.01^C^	53.27 ± 0.48^B^
	Mean Square	19.01**	0.31	0.052***	2.38***	0.41***	27.83***
	t Value	−21.93	−0.79	−0.50	−2.08	2.79	14.09
	Pr > |t|	<0.0001	0.4590	0.6326	0.0826	0.0317	<0.0001

Values presented as mean ± standard deviation.

Means in the same column with different alphabets in superscript are significantly different (*p* ≤ 0.001).

The means shown in the same column with common superscripts are not significantly different (*p* > 0.05).

***Highly significant at 0.001.

The significantly higher content of minerals such as K (1929.04 ppm) and P (4188.85 ppm) was noticed in IQMH 203 and MCFL 15 genotypes, respectively (Table 4). Mineral contents such as copper (2.21–2.36 ppm), zinc (37.05–52.40 ppm), calcium (410–590 ppm), and potassium (2,915–3,471 ppm) were also reported in earlier studies (38). Similar results for Zn content (30.51–42.18 ppm) in maize varieties were also observed by Kabir et al. (39). The difference observed in the mineral composition might be due to the varietal difference, environmental effect, or type of irrigation or fertilizer used. Vaswani et al. (36) stated that the genotypic effect is more prominent in the composition than other environmental factors. The mineral content of *chapatti* revealed that cooking greatly affects the composition of the minerals. Zn, Cu, Mn, P, Ca, and K contents of *chapatti* varied from 38.10 to 46.30 ppm; 2.05–3.51 ppm; 4.50-7.40 ppm; 3,156.85–4,128.35 ppm; 219.46–491 ppm; and 1,546.72–1,942.55 ppm, respectively. It was observed that the *chapatti* samples had significantly higher Zn, Cu, Mn, Ca, and K contents except for P in comparison to flour samples. It might be due to the cooking process involved during the preparation of *chapatti*. The Zn (46.30 ppm) and Ca (491 ppm) contents of *chapatti* prepared from PMH 10 were much higher.

**TABLE 4:** Mineral and amino acid content of maize flours and *chapatti* prepared from different genotypes.

Flour	Genotypes	Mineral content (ppm)						Amino acid content (gm/100g protein)	
			
		Zn	Cu	Mn	P	Ca	K	Lysine	Tryptophan
1	PMH 10	38.25 ± 0.23^B^	2.69 ± 0.09^B^	6.69 ± 0.05^B^	3478.35 ± 3.08^E^	321.46 ± 1.82^A^	1552.55 ± 1.62^F^	1.77 ± 0.08^CD^	0.44 ± 0.02^CD^
2	IQPMH 1708	28.85 ± 0.11^C^	2.15 ± 0.03^CD^	3.90 ± 0.03^G^	3459.87 ± 3.04^F^	58.51 ± 1.26^E^	1917.05 ± 1.02^B^	4.28 ± 0.14^B^	1.07 ± 0.04^B^
3	IQMH 203	35.60 ± 0.06^B^	2.65 ± 0.04^B^	5.55 ± 0.03^D^	4019.35 ± 3.05^B^	55.00 ± 3.60^E^	1929.04 ± 1.71^A^	4.76 ± 0.21^A^	1.19 ± 0.05^A^
4	MCFL 15	37.10 ± 1.73^B^	2.05 ± 0.05^D^	5.05 ± 0.02^E^	4188.85 ± 2.73^A^	133.33 ± 3.78^B^	1753.54 ± 1.04^C^	1.64 ± 0.11^CD^	0.41 ± 0.02^CD^
5	MCFL 346	30.30 ± 0.11^C^	2.30 ± 0.14^C^	6.45 ± 0.03^C^	3589.52 ± 2.72^D^	92.66 ± 4.04^C^	1654.54 ± 2.07^E^	1.84 ± 0.17^C^	0.46 ± 0.04^C^
6	White Hybrid 574	42.77 ± 5.70^A^	1.55 ± 0.03^E^	4.45 ± 0.05^F^	3730.85 ± 5.32^C^	83.00 ± 2.64^D^	1524.21 ± 2.99^G^	1.53 ± 0.12^DE^	0.38 ± 0.03^DE^
7	Landrace 593	37.80 ± 0.08^B^	3.21 ± 0.20^A^	7.10 ± 0.07^A^	3736.35 ± 3.50^C^	92.50 ± 1.97^C^	1728.54 ± 2.67^D^	1.39 ± 0.08^E^	0.35 ± 0.02^E^
	Mean Square	69.39***	0.86***	4.34***	224549.98***	25821.60**	77164.67***	5.79***	0.36***
***Chapatti***									
1	PMH10	46.30 ± 0.16^A^	2.71 ± 0.06^C^	6.71 ± 0.04^B^	3339.35 ± 2.05^F^	491.00 ± 4.35^A^	1586.55 ± 3.58^E^	1.64 ± 0.11^B^	0.41 ± 0.02^B^
2	IQPMH 1708	41.05 ± 0.19^C^	3.20 ± 0.04^AB^	4.50 ± 0.02^G^	3156.85 ± 3.60^G^	360.55 ± 6.23^B^	1931.22 ± 13.79^A^	4.12 ± 0.07^A^	1.03 ± 0.02^A^
3	**IQMH 203**	46.00 ± 0.22^A^	3.00 ± 0.03^BC^	6.60 ± 0.02^C^	3773.85 ± 4.39^B^	293.66 ± 6.42^D^	1942.55 ± 3.21^A^	4.44 ± 0.12^A^	1.11 ± 0.03^A^
4	MCFL 15	39.31 ± 0.58^D^	3.40 ± 0.05^A^	6.10 ± 0.04^E^	4128.35 ± 5.56^A^	333.00 ± 2.64^C^	1823.05 ± 3.59^B^	1.40 ± 0.14^C^	0.35 ± 0.04^C^
5	MCFL 346	38.10 ± 0.12^E^	2.26 ± 0.28^D^	6.48 ± 0.03^D^	3585.18 ± 2.98^D^	254.00 ± 3.00^E^	1701.04 ± 2.62^D^	1.64 ± 0.08^B^	0.41 ± 0.02^B^
6	White Hybrid 574	45.10 ± 0.08^B^	2.05 ± 0.06^D^	4.74 ± 0.03^F^	3629.35 ± 3.54^C^	219.46 ± 2.70^F^	1546.72 ± 16.65^F^	1.80 ± 0.11^B^	0.45 ± 0.02^B^
7	Landrace 593	41.45 ± 0.23^C^	3.51 ± 0.44^A^	7.40 ± 0.05^A^	3406.84 ± 2.60^E^	226.66 ± 4.16^F^	1763.05 ± 2.54^C^	1.68 ± 0.11^B^	0.42 ± 0.02^B^
	Mean Square	33.07***	0.95***	3.43***	303723.50***	27189.75***	72787.89***	5.36***	0.33***
	t Value	−4.42	−2.61	−2.90	3.57	−8.33	−4.47	0.25	0.24
	Pr > |t|	0.0045	0.0400	0.0273	0.0117	0.0002	0.0042	0.8107	0.8211

Values presented as mean ± standard deviation.

Means in the same column with different alphabets in superscript are significantly different (*p* ≤ 0.001).

The means shown in the same column with common superscripts are not significantly different (*p* > 0.05).

***Highly significant at 0.001.

Lysine and tryptophan contents of maize flours and *chapattis* are summarized in Table 4. The lysine and tryptophan contents were observed to be in the range of 1.39–4.76 g and 0.35–1.19 g per 100 g of protein and significantly differed among various genotypes. IQMH 203 showed a higher value of lysine followed by IQPMH 1708. Landrace 593 showed the lowest lysine content which is 1.39 g/100 g of protein. A similar trend in amino acid content was observed in *chapatti* prepared from different genotypes. The lysine and tryptophan contents of *chapatti* prepared from QPM hybrids, *viz*., IQPMH 1708 and IQMH 203 were recorded to be 4.12 and 1.03, and 4.44 and 1.11 g/100 g, respectively. However, the lysine and tryptophan contents in *chapatti* were lower as compared to flours which could be due to the effect of baking conditions (40). A study by Gallego-Castillo et al. (41) in non-QPM and QPM-based processed products, namely tortillas, arepas, and mazamorra, showed a true retention value of tryptophan content that is 62.27, 16.67, 15.91%, and 66.29, 23.44, and 19.69%, respectively. During processing, the reduction in lysine content might be due to the occurrence of the Maillard reaction, which modifies the starch and protein structures and leads to more availability of reducing sugars and reactive sites of protein, respectively (42). It was also found that the lysine and tryptophan contents are more than double in the QPM-based *chapattis* as compared to normal and landrace genotypes-based *chapattis*. Hence, *chapattis* prepared from QPM genotypes are more nutritious and beneficial for human consumption than chapattis prepared from normal maize.

### Color parameter of *chapatti*

Hunter color laboratory parameters such as L*, a*, and b* values among flours and chapattis prepared from different maize genotypes were observed (Table 5). The L* value of flour and *chapatti* from different maize genotypes significantly varied from 84.88 to 90.63 and 47.09–74.96, respectively. In the case of flour and *chapattis*, the highest L * value (lightness) was observed for MCFL 346 and the lowest for PMH 10. L* values of IQPMH 1708 and IQMH 203 were observed to be 87.38 and 89.18 indicating that the color of *chapattis* was acceptable and preferred by the consumer. L* value of 81.94 to 86.96 for corn flours from various genotypes have been reported by Sandhu et al. (37). Kathuria et al. (43) analyzed the color value of maize flour to be around 70.05 ± 0.02. The a* value presents the redness or greenness which ranged from −0.57 to 1.48 and 2.25–5.31 in flour and chapatti, respectively. The highest a* and b* values in PMH 10-based flour and *chapatti* might be due to the high level of anthocyanins and carotenoids, respectively (17). ΔL, Δa, Δb, and ΔE values indicate the color difference for lightness, redness-greenness, blueness-yellowness, and total color difference, respectively, for different genotypes-based corn flour and these values ranged from −13.99 to −8.24, −0.43 to 1.62, 8.15 to 22.19, and 12.16 to 26.28, respectively. Genotypes, *viz*., White Hybrid 574, MCFL 15, and MCFL 346 exhibited no significant difference in total color difference value. IQMH 203 and IQPMH 1708 were not significantly different with respect to the b* value in maize flours. Highly significant differences for parameters such as L (8.46), a*(−9.18), b* (−12.54), ΔL (9.83), Δa (−10.35), Δb (−9.61), and ΔE (−10.84) were observed among corn flours and *chapatti* for different genotypes.

**TABLE 5:** Color analysis of maize flour and *chapatti* from different genotypes.

Flour	Genotypes	L*	a*	b*	ΔL	Δa	Δb	ΔE
1	PMH 10	84.88 ± 0.27^C^	1.48 ± 0.11^A^	21.85 ± 0.85^A^	−13.99 ± 0.27^C^	1.62 ± 0.11^A^	22.19 ± 0.85^A^	26.28 ± 0.84^A^
2	IQPMH 1708	87.38 ± 2.62^BC^	−0.18 ± 0.05^C^	14.64 ± 0.61^B^	−11.55 ± 2.61^BC^	−0.18 ± 0.10^CD^	14.98 ± 0.61^B^	18.98 ± 1.26^B^
3	IQMH 203	89.18 ± 0.48^AB^	−0.57 ± 0.03^E^	13.93 ± 0.22^B^	−9.69 ± 0.48^AB^	−0.43 ± 0.03^E^	14.28 ± 0.22^B^	17.27 ± 0.18^C^
4	MCFL 15	88.37 ± 1.79^AB^	−0.39 ± 0.05^D^	7.87 ± 0.73^D^	−10.49 ± 1.77^AB^	−0.25 ± 0.04^D^	8.21 ± 0.73^D^	13.38 ± 1.08^D^
5	MCFL 346	90.63 ± 0.15^A^	−0.25 ± 0.03^C^	9.91 ± 0.41^C^	−8.24 ± 0.15^A^	−0.14 ± 0.03^C^	10.25 ± 0.41^C^	13.16 ± 0.24^D^
6	White Hybrid 574	89.85 ± 0.38^AB^	−0.39 ± 0.06^D^	7.81 ± 0.57^D^	−9.02 ± 0.38^AB^	−0.25 ± 0.06^CD^	8.15 ± 0.57^D^	12.16 ± 0.16^D^
7	Landrace 593	87.31 ± 2.33^BC^	0.22 ± 0.04^B^	10.43 ± 0.15^C^	−11.56 ± 2.33^BC^	0.36 ± 0.04^B^	10.77 ± 0.15^C^	15.85 ± 1.72^C^
	Mean Square	11.04***	1.48***	74.11***	11.10***	1.52***	74.12***	71.10***
***Chapatti***								
1	PMH 10	47.09 ± 1.88^E^	5.31 ± 0.11^A^	32.23 ± 2.11^A^	−48.47 ± 6.69^C^	5.43 ± 0.13^A^	33.24 ± 2.63^A^	61.56 ± 1.00^A^
2	IQPMH 1708	61.78 ± 2.32^D^	5.23 ± 1.61^A^	21.11 ± 0.42^B^	−37.09 ± 2.32^B^	5.37 ± 1.61^A^	21.43 ± 0.42^B^	43.20 ± 2.02^B^
3	IQMH 203	63.10 ± 1.38^CD^	4.68 ± 0.58^AB^	21.20 ± 1.01^B^	−35.77 ± 1.38^B^	4.82 ± 0.58^AB^	21.54 ± 1.01^B^	42.06 ± 0.89^B^
4	MCFL 15	69.89 ± 1.67^B^	3.76 ± 1.66^ABC^	17.63 ± 0.81^CD^	−32.65 ± 6.83^B^	3.24 ± 1.65^C^	17.97 ± 0.81^CD^	34.20 ± 1.65^D^
5	MCFL 346	74.96 ± 1.73^A^	3.60 ± 0.54^ABC^	18.42 ± 1.54^BC^	−29.84 ± 1.97^AB^	3.75 ± 0.54^BC^	18.78 ± 1.54^BC^	35.40 ± 2.42^CD^
6	White Hybrid 574	68.83 ± 2.05^B^	2.25 ± 0.79^C^	15.29 ± 2.69^D^	−22.92 ± 2.76^A^	2.98 ± 1.18^C^	15.63 ± 2.69^CD^	28.61 ± 2.93^E^
7	Landrace 593	66.82 ± 4.86^BC^	2.70 ± 1.9^BC^	16.25 ± 1.40^CD^	−31.92 ± 4.64^B^	3.17 ± 1.94^C^	15.26 ± 2.78^D^	37.98 ± 2.51^C^
	Mean Square	237.50***	4.32***	97.95***	184.08***	3.45***	112.34***	333.34***
	t Value	8.46	−9.18	−12.54	9.83	−10.35	−9.61	−10.84
	Pr > |t|	0.0001	<0.0001	<0.0001	<0.0001	<0.0001	<0.0001	<0.0001

Values presented as mean ± standard deviation.

Means in the same column with different alphabets in superscript are significantly different (*p* ≤ 0.001).

The means shown in the same column with common superscripts are not significantly different (*p* > 0.05).

***Highly significant at 0.001.

### Textural properties of *chapatti*

The textural properties directly affect the overall acceptability of *chapatti* (20). The results indicated that the different maize genotypes exhibited significant differences in the shear force of the *chapatti*. The shear force value is mainly related to the freshness and pliability of the final product. The value of shear force was found to be in the range of 3.36 (Landrace 593) − 6.96 N (White hybrid 574), (Table 6). A decrease in shear force resulted in an increase in pliability and soft texture which might be due to the higher retention of moisture in c*hapatti* (20).

**TABLE 6:** Sensory attributes and textural properties (Shear Value) of *Chapatti* from different genotypes.

	Sensory attributes					Textural properties
		
	Genotypes	Color	Aroma	Taste	Overall acceptability	Shear value (N)
1	PMH 10	6 ± 0.00^AB^	6.3 ± 1.15^A^	6.3 ± 0.58^AB^	6 ± 0.5^A^	4.77 ± 0.52^C^
2	IQPMH 1708	7.3 ± 0.58^A^	7 ± 0.00^A^	7 ± 1.00^A^	7 ± 0.4^A^	3.73 ± 0.43^D^
3	IQMH 203	7.7 ± 1.53^A^	6 ± 1.73^AB^	7 ± 1.00^A^	7 ± 1.4^A^	4.03 ± 0.42^CD^
4	MCFL 15	5 ± 1.00^BC^	4.3 ± 1.15^BC^	4.7 ± 0.58^C^	5 ± 0.3^B^	3.57 ± 0.28^E^
5	MCFL 346	6.3 ± 1.53^AB^	3.3 ± 1.15^C^	5 ± 1.00^BC^	5 ± 0.4^B^	5.98 ± 0.77^B^
6	White Hybrid 574	4 ± 1.73^C^	5.3 ± 0.58^AB^	5.7 ± 0.58^ABC^	5 ± 0.3^B^	6.96 ± 0.73^A^
7	Landrace 593	4 ± 0.00^C^	5.3 ± 0.58^AB^	5.7 ± 1.53^ABC^	5 ± 0.6^B^	3.36 ± 0.21^D^
	Mean Square	6.63***	4.60***	2.52**	3.19***	6.73***

Values presented as mean ± standard deviation.

Means in the same column with different alphabets in superscript are significantly different (*p* ≤ 0.001).

The means shown in the same column with common superscripts are not significantly different (*p* > 0.05).

***Highly significant at 0.001.

### Sensory attributes of maize-based *chapatti*

The sensory score of *chapatti* made from various maize genotypes is elucidated in Table 6. Maize *chapatti* prepared from IQMH 203 and IQPMH 1708 was rated highest in terms of color, taste, aroma, and overall acceptability and were not significantly different from each other. *Chapatti* prepared from White Hybrid 574 and Landrace 593 was not highly acceptable in terms of sensory attributes. Hence, due to the relatively higher sensory score of IQMH 203 and IQPMH 1708 coupled with their relative nutritional value in terms of mineral profile and essential amino acids, they were considered the most appropriate varieties for the production of maize-based nutritious flat breads.

### Differentiation of QPM *chapattis* from normal maize

Figure 2 provides a schematic representation of the mechanism, by which QPM results in higher protein quality as compared to normal maize. *The opaque-2* gene positively regulates low-quality prolamin proteins in normal maize, whereas its mutation in QPM increases higher-quality non-prolamin proteins, including albumins, globulins, and glutelins. By virtue of its replacement by higher quality non-prolamins, the lower expression of prolamins increases protein quality on one hand and decreases the chances of any adverse reactions in some patients with celiac disease as observed in normal maize (44). In order to enable commercialization of the biofortified products in the market, it is necessary to employ a rapid method for Quality Control and consumer empowerment. Using a previously standardized process (Indian patent applied), we quantified protein quality in *chapattis* made from normal maize and QPM. The samples were read at 595 nm after processing. A lower value indicates less nutritionally poor protein fraction, thereby higher overall maize protein quality. Conversely, a higher amount of nutritionally poor protein indicates overall lower maize protein quality. The readings of IQPMH 1708 and IQMH 203 at 595 nm were 0.135 and 0.152, respectively, while the readings for normal genotypes were above 0.25. This indicates that a cut-off of 0.2 at 595 nm is indicative of protein quality in maize chapattis. This process requires less than 10 min to complete, providing a good tool for the quality control of the product.

## Conclusion

With respect to modern lifestyles and healthy eating trends, traditional and nutritional food products are gaining popularity. *Chapatti* is a major staple baked food in most households and could bring the combination of nutrition and goodness of maize. Hence, the present study was executed for a better understanding of the nutritional and chapatti-making quality attributes of different maize genotypes. C*hapatti*s prepared from QPM showed higher lysine and tryptophan content as compared to other genotypes. The overall quality score of *chapatti* prepared from IQMH 203 and IQPMH 1708 scored higher and imparted a desirable aroma coupled with *chapatti* of better texture, taste, and acceptability. Therefore, such cultivars need to be popularized for nutritional security at low cost in midday meals and other nutrition schemes of the government as well as for catering to patients with celiac disease. Given the listing of maize *chapatti* in traditional delicacies, there is ample scope for entrepreneurship development in this sector using QPM. The availability of a rapid protocol to differentiate the products made from QPM from those of normal maize is an added advantage to ensure quality control and empower consumers. Overall, the study provides a comparative assessment of different maize types for *chapatti*-making and shows the ability of rapid differentiation to categorize and confirm the final product based on protein quality.

## Data availability statement

The original contributions presented in this study are included in the article/supplementary material, further inquiries can be directed to the corresponding author.

## Author contributions

NK performed the experiments and prepared the manuscript. RK planned the experiments and reviewed the manuscript. AS performed the experiments on Rapid Kit and reviewed the manuscript. DS and DC reviewed the manuscript. BS planned the experiments. AD analyzed the results. PK and YK performed the experiments. PS performed the mineral content analysis. All authors contributed to the article and approved the submitted version.

## Footnotes

1
https://www.fao.org/faostat/en

2
https://punjab.gov.in/culture/
